# Overcoming doxorubicin resistance of cancer cells by Cas9-mediated gene disruption

**DOI:** 10.1038/srep22847

**Published:** 2016-03-10

**Authors:** Jong Seong Ha, Juyoung Byun, Dae-Ro Ahn

**Affiliations:** 1 The Center for Theragnosis, Biomedical Research Institute, Korea Institute of Science and Technology, Hwarangro 14-gil 5, Seongbuk-gu, Seoul 136-791, Republic of Korea; 2 Department of Biological Chemistry, KIST School, University of Science and Technology (UST), Hwarangro 14-gil 5, Seongbuk-gu, Seoul 136-791, Republic of Korea

## Abstract

In this study, Cas9 system was employed to down-regulate *mdr1* gene for overcoming multidrug resistance of cancer cells. Disruption of the MDR1 gene was achieved by delivery of the Cas9-sgRNA plasmid or the Cas9-sgRNA ribonucleoprotein complex using a conventional gene transfection agent and protein transduction domain (PTD). Doxorubicin showed considerable cytotoxicity to the drug-resistant breast cancer cells pre-treated with the RNA-guided endonuclease (RGEN) systems, whereas virtually non-toxic to the untreated cells. The potency of drug was enhanced in the cells treated with the protein-RNA complex as well as in those treated with plasmids, suggesting that mutation of the *mdr1* gene by intracellular delivery of Cas9-sgRNA complex using proper protein delivery platforms could recover the drug susceptibility. Therefore, Cas9-mediated disruption of the drug resistance-related gene can be considered as a promising way to overcome multidrug resistance in cancer cells.

Cancer cells become resistant to one or more anticancer drugs after repeated treatment of chemotherapeutic agents^1^. This multidrug resistance (MDR) is a main hurdle to achieve successful cancer therapy. There have been various mechanisms suggested to explain MDR, which include altering membrane transport protein to increase drug efflux, enhancing DNA repair, amending cell cycle regulation to block apoptosis, and detoxification^2^,^3^. Discovery of agents that efficiently overcome MDR with reduced toxicity has been the focus of extensive research^4^,^5^,^6^,^7^. A traditional approach is to develop inhibitors of MDR1 (or P-gp) protein, a membrane-bound active drug efflux pump, responsible for one of the most important mechanisms involved in many MDR cells^8^,^9^. However, laborious and costly chemical screening procedures are required to find potent MDR1 inhibitors^10^. Another way to overcome MDR is to deliver drugs using pathways that are independent from the efflux protein^5^,^11^. Nanocarriers have been effective particularly in this approach utilizing efficient cellular uptake of the drug-loaded nanoparticles and subsequent rapid release of a large amount of the anticancer drug to induce cytotoxicity. As another alternative way to control the protein, down-regulation of *mdr1* gene by gene regulation technology such as siRNA and virus-mediated gene modulation has also been considered^12^,^13^. The siRNA method is a temporal way to lower the *mdr1* mRNA level, requiring continuous treatments for consistent down-regulation of the gene. In contrast, the *mdr1* DNA can be knocked out permanently by viral vector-based gene regulation, which, however, still has potential risks stemming from virus-derived toxicity and immunogenicity^14^,^15^.

Recently, CRISPR (clustered regularly interspaced short palindromic repeats)-associated protein-9 nuclease (Cas9) has become a very popular tool for gene modification^16^. Cas9 protein targets a double stranded DNA gene complementary to the recognition sequence of the single-stranded guide RNA (sgRNA) complexed in the protein and breaks the DNA, leading to disruption of the gene^17^,^18^. In contrast with siRNA, Cas9 can permanently down-regulate the target gene, providing an efficient strategy for gene therapy. Due to its relatively simple manipulation protocol, Cas9-based genome engineering has quickly proven to be a useful tool for screening functional genome and creating disease animal models^19^,^20^. In this study, we attempted to reduce the drug resistance in cancer cells by utilizing the Cas9-sgRNA system targeting the *mdr1* gene (Fig. 1). To edit the target gene, we transfected the cells with the Cas9-guide RNA (gRNA) plasmid. In addition, we also investigated alternative approaches to introduce the RNA-guided endonuclease (RGEN) system in the cells, intracellularly delivering the RNA-protein (RNP) complex prepared *in vitro* by using two different carriers. Finally, doxorubicin (DOX) was treated to the cells after *mdr1* was disrupted by the Cas9-sgRNA system to examine the recovered drug sensitivity of the cells by the gene editing tool.

## Results

**Figure 2:**
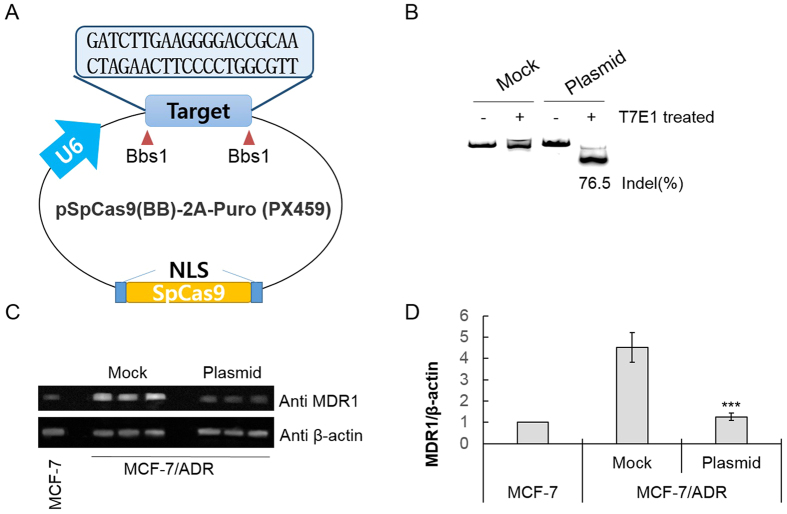
Disruption of mdr1 using Cas9-sgRNA plasmid. (**A**) The pSpCas9(BB)-2A-Puro plasmid containing Cas9 and sgRNA scaffolds. The target sequence for *mdr1* was inserted into the plasmid using BbsI sites. (**B**) The T7E1 cleavage assay to detect disruption in the target gene. (**C**) Western blot showing MDR1 protein levels in MCF-7/ADR cells transfected with the mock vector and the Cas9-sgRNA plasmid from three independent experiments. The protein level of the drug-sensitive MCF cells was also compared. (**D**) Densitometric analysis of the Western blot was performed for quantitative evaluation of the protein level after normalization by the expression level of β–actin. The data represent the mean ± s.d. (n = 3) ***P ≤ 0.001 vs. the mock vector-treated MCF-7/ADR cell.

The sgRNA was designed to target the coding sequence for N-terminal part of MDR1 protein that contains protospacer adjacent motif (PAM) using Guide RNA Target Design Tool (Blue Heron, USA) (Table S1). Then, we cloned the target sequence into the Cas9-expressing plasmid pSpCas9(BB)-2A-Puro (Fig. 2A) and transfected the resulting construct (2 μg) in DOX-resistant breast cancer cells (MCF7/ADR). The target region was amplified by PCR and treated with T7 endonuclease 1 (T7E1) which can cleave the mismatched sequences produced due to insertion and deletion mutations (indels) after the DNA breaks by Cas9. Gel analysis demonstrated 76.5% disruption of the target region in the transfected cells (Fig. 2B). When the expression level of the MDR1 protein influenced by the gene disruption was examined by Western blotting, it was lowered to 25% relative to that in the untreated cells (Fig. 2C,D). Treatment with higher amount of the plasmid (3 μg) did not significantly down-regulate the MDR1 protein expression (Fig. S1).

After observing disruption of *mdr1* by transfection of the cells with the Cas9-sgRNA plasmid, we tested whether the cellular uptake of the RNP complex prepared *in vitro* could also lead to disruption of the target gene. Recombinant Cas9 was bacterially expressed and purified following the literature procedures (Fig. S2)^21^. The sgRNA targeting *mdr1* was prepared by means of *in vitro* transcription (Fig. S3). The 2:1 ratio between the protein and the RNA was adopted for the preparation of RNP complex after screening *in vitro* double stranded break (DSB) activity at various binding ratios (Fig. S4).

**Figure 3:**
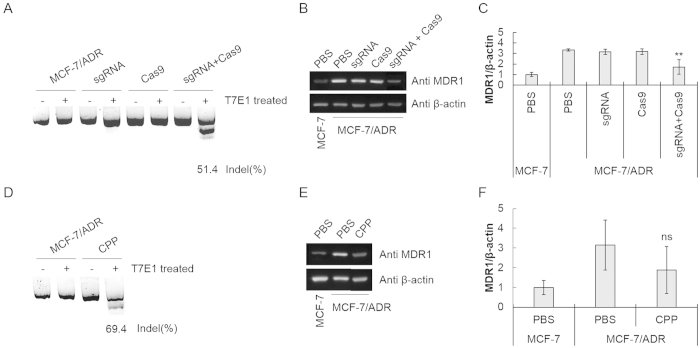
Disruption of mdr1 by intracellular delivery of the Cas9-sgRNA ribonucleocomplex. (**A**) The T7E1 cleavage assay to detect the gene disruption and (**B**) Western blot to measure MDR1 protein levels in MCF-7/ADR cells treated with the Cas9-sgRNA ribonucleocomplex using LF3K. (**C**) Densitometric analysis of the Western blot was performed for quantitative evaluation of the protein level after normalization by the expression level of β–actin. The data represent the mean ± s.d. (n = 3). **P ≤ 0.01 vs. PBS-treated MCF-7/ADR cell. (**D**) The T7E1 cleavage assay to detect the gene disruption and (**E**) Western blot to measure MDR1 protein levels in MCF-7/ADR cells treated with the Cas9-sgRNA ribonucleocomplex using TAT. (**F**) Densitometric analysis of the Western blot was performed for quantitative evaluation of the protein level after normalization by the expression level of β–actin. The data represent the mean ± s.d. (n = 3). ns *P* > 0.05 vs. PBS-treated MCF-7/ADR cell.

Two different carrier systems such as Lipofectamine 3000 (LF3K), a commercially available cationic lipid and TAT, a well-known protein transduction domain (PTD) were used for intracellular delivery of the complex. When the complex was delivered by using LF3K, the gene disruption efficiency reached to 51.4% (Fig. 3A). The corresponding protein level was decreased by 64% as analyzed in Western blotting (Fig. 3B,C). The gene disruption and the corresponding down-regulation of the target protein were not observed when sgRNA or Cas9 protein alone was treated. For delivery of the complex using PTD, Cas9 was conjugated covalently with TAT by a transglutaminase reaction using excess amount of the peptides by following the known protocol^22^. The unreacted peptides were not removed but left for their physical conjugation with sgRNA in accordance with the previously reported procedure for PTD-mediated Cas9 delivery^18^. The TAT-RNP complex prepared by mixing the TAT-protein conjugate and the RNA with 2:1 ratio. PTD-mediated intracellular delivery of the complex resulted in 69.4% gene disruption in the drug resistant cells and 45% decrease of the MDR1 protein expression, displaying similar levels of gene and protein regulation to those showed by the complex delivered by LF3K (Fig. 3D–F). When compared with the plasmid, both carrier systems for delivery of the RNP complex generated slightly lower cell populations with the damaged *mdr1.*

To additionally examine indel patterns of the disrupted mdr1 gene by using the conventional Sanger sequencing method, we extracted genomic DNA of the cells treated either with the plasmid or the RNP complex, and the target gene was amplified by PCR. After transformation of the amplicons was performed, plasmids from 50 colonies in each method were sequenced for indel analysis. Although the disruption rates were lower (~30%) than those observed in T7E1 assays, we clearly found that indels in the targeting region. The gene was mainly disrupted with deletion by both RNP complex- and plasmid-based methods (Fig. S5). We also tested off-targeting rates of the gene disruption methods on two non-target regions sharing considerable common sequences with the target area. Sequencing data revealed that no disruption was observed in the non-target area (Fig. S6).

**Figure 4:**
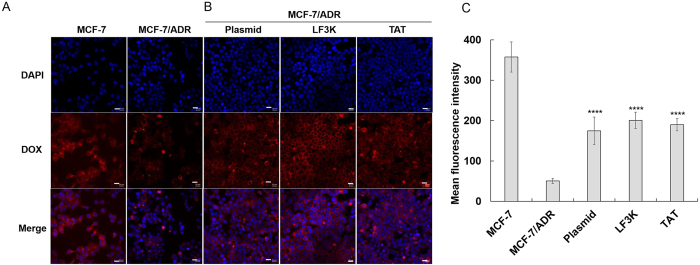
Intracellular accumulation of DOX in the drug-resistant cells after mdr1 gene disruption by Cas9-sgRNA. (**A**) Fluorescence microscopic images showing DOX accumulation in MCF-7 (drug-sensitive) and MCF-7/ADR (drug-resistant) cells. (**B**) Fluorescence microscopic images showing DOX accumulation in MCF-7/ADR cells treated with Cas9-sgRNA (scale bar: 20 μm). (**C**) Flow cytometric analysis of intracellular DOX accumulation. The data represent the mean ± s.d. (n = 12). ****P ≤ 0.0001 vs. untreated MCF-7/ADR cells.

After demonstrating that the gene for drug resistance and the relevant protein could be down-regulated by Cas9-sgRNA, we investigated potency of DOX in the MCF7/ADR cells pre-treated with Cas9-sgRNA. For the drug delivery study, the accumulation of DOX were initially analysed by fluorescence microscopy. When treated with DOX, the MCF-7/ADR cells showed relatively low drug accumulation compared to that of the wild-type MCF-7 cell line (Fig. 4A). Substantially increased amount of the drug, however, was delivered into the drug-resistant cells transfected with the Cas9-sgRNA plasmid (Fig. 4B, left). Similarly, when the RNP complex instead of the plasmid was delivered by LF3K or TAT into the drug-resistance cells, the cells also showed higher intracellular drug levels compared with the untreated MCF-7/ADR cells (Fig. 4B, middle and right). These results proved that Cas9-sgRNA-mediated disruption of *mdr1* lowered MDR1 protein expression and subsequently decreased the drug efflux based on the membrane protein, thereby leading to intracellular accumulation of the drug. When analysed by flow cytometry for more quantitative comparison of the drug uptake, RNP and plasmid-treated cells showed comparable intracellular accumulation of DOX, which was four-fold increased drug uptake relative to that in the untreated cells (Fig. 4C). However, this increased drug uptake level was still lower than that of the drug-sensitive cells (MCF-7).

**Figure 5:**
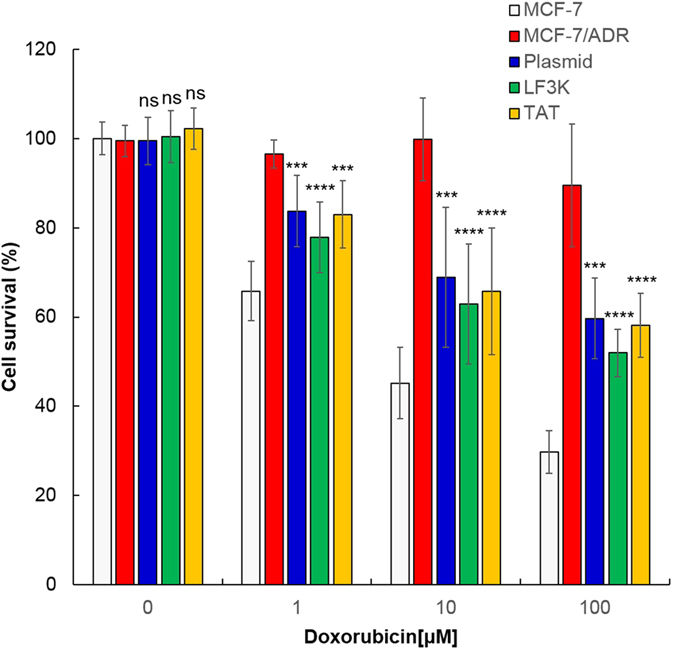
Enhanced potency of DOX in the drug-resistant cells after mdr1 gene disruption by Cas9-sgRNA. Cell viability of MCF-7/ADR cells at various concentrations of DOX was examined after treatment with Cas9-sgRNA using the plasmid (blue bars), LF3K (green bars) and TAT (yellow bars), and compared with that of untreated MCF-7 (white bars) and MCF-7/ADR cells (red bars). The data represent the mean ± s.d. (n = 7). ns *P* > 0.05, ***P ≤ 0.001, ****P ≤ 0.0001 vs. untreated MCF-7 cells.

Finally, we tested cytotoxicity of DOX in the cells of which *mdr1* is edited by Cas9-sgRNA. In the cell viability test, DOX exhibited considerable cytotoxicity in the drug-resistance breast cancer cells transfected with the Cas9-sgRNA plasmid (Fig. 5, blue bars), whereas the anticancer agent caused no significant cytotoxicity in untreated MCF7/ADR cells (Fig. 5, red bars). In the cells treated with the RNP complex, the drug showed the similar potency at the same concentration, compared with that in the plasmid-treated cells. (Fig. 5, green and orange bars) The increase of drug potency was not observed in the untreated cells and the cells treated with the protein or RNA alone due to inability to disrupt the target gene (Fig. S7). The three ways to disrupt *mdr1* tested (the Cas9-sgRNA plasmid, the Lipofectamine/RNP complex, and the TAT-RNP complex) yielded the similar recovery of the drug sensitivity by gene edition, resulting in 50–60% cell viability after treatment of 100 μM DOX. However, this drug potency was still lower than that found in the drug-sensitive MCF7 although the drug-resistance was nearly reversed at lower concentrations of DOX (0.1–10 μM). Nevertheless, these results highlight that Cas9-mediated disruption of the drug-resistant gene can be used to overcome the drug resistance in cancer cells.

## Discussion

Drug resistance is a major challenge in chemotherapy. Development of new drugs that are not subject to the resistance mechanism requires enormous time and cost, and can be inactive after resistance to the drugs gained in the cancer cells. Alternatively, blocking the resistance mechanisms would allow to use the already existing anticancer agents, suggesting an efficient therapeutic strategy. In this study, we report an approach using Cas9-mediated disruption of *mdr1*, the responsible gene for effluxing the internalized anticancer drug in cells, as a potential way to overcome the drug resistance in cancer cells. We used three different ways such as transfection of Cas9-sgRNA plasmid, Lipofectamine-mediated delivery of the RNP complex, and TAT-mediated delivery of the complex to introduce the Cas9 system into drug resistant breast cancer cells. Although transfecting cells with the plasmid is the conventional way to execute gene editing in the Cas9 system, delivery of the RNP complex also provided considerable efficiencies in disruption of the target gene. This is consistent with the previous study reporting that delivery of RNP complex yielded as high indels as transfection of the plasmid expressing the complex^23^. Potency of DOX in the MDR cells was improved after treatment of the cells with Cas9-sgRNA, because disruption in *mdr1* reversed the drug resistance. Additional treatments of Cas9-gRNA plasmid did not increase potency of the DOX (Fig. S8), implicating that the incomplete reversal of the drug-resistance is due not only to the matter of delivery efficiency but also to MDR1-independent drug resistance^24^,^25^. The *mdr1*-derived drug resistance can be controlled at genomic DNA, mRNA, and protein levels. While modulators acting on protein and mRNA levels suppress temporally the drug resistance of the cells^14^,^26^, edition of chromosomal DNA remove the resistance irreversibly. Since MDR1 is also actively expressed in normal tissues responsible for detoxification such as liver^3^, treatment of Cas9-sgRNA for disruption of the *mdr1* gene should be pursued by utilizing tumor-targeted drug delivery systems^27^. Based on this strategy, we expect that engineering genes related with the drug resistance using Cas9-sgRNA system will provide a novel way to overcome the multidrug resistance of cancer cells and thereby have immense potentials in cancer therapeutics.

## Methods

### Materials

*MagListo*™ 5 M Genomic DNA Extraction Kit and all nucleic acids were purchased from Bioneer (Korea). The pMJ806 and pSpCas9 (BB)-2A-Puro (PX459) plasmids were provided by Addgene (plasmid # 39312 and plasmid # 48139, repectively)^21^,^26^. T7 Endonuclease, Bbs1 endonuclease and HiScribe™ T7 High Yield RNA Synthesis Kit were purchased from New England Biolabs (USA). Power *Pfu* polymerase (ver 2.0) was purchased from NanoHelix (Korea). Lysozyme, doxorubicin-HCl (DOX), transglutaminase, imidazole, protease inhibitor cocktail (PIC), phenylmethylsulfonyl fluoride (PMSF), absolute ethanol and isoamyl alcohol were purchased from Sigma-Aldrich (USA). Rosetta™ 2(DE3) Competent Cells were purchased from Novagen (Germany). All buffers were obtained from Biosesang (Korea). SYBR® Gold Nucleic Acid Gel Stain, SuperSignal™ West Pico Chemiluminescent Substrate and HisPur^TM^ Ni-NTA Resin were purchased from Thermo Fisher Scientific (USA). TEV protease was expressed and purified following protocol^28^. Amicon Ultra-15 Centrifugal Filter Unit with Ultracel-100 membrane (MWCO 100 K) was obtained from Merck Millipore (USA). Bacto^TM^ Yeast Extract and Bacto^TM^ Tryptone were purchased from BD Biosciences (USA). RPMI, DMEM, FBS and antibiotics (1% penicillin and 1% streptomycin) were purchased from Gibco (USA). Lipofectamine 3000 (LF3K) was purchased from Invitrogen (USA). Well plates were purchased from SPL Lifesciences (Korea). RIPA lysis buffer was purchased from ATTO (Japan). All Antibodies were purchased from Abcam (UK). CCK-8 assay kit was purchased from Dojindo Molecular Technologies (Japan). DPBS was purchased from Welgene (Korea). Isopropyl β-D-1-thiogalactopyranoside (IPTG) was purchased from Biobasic (Canada). NaCl was purchased from Daejung (Korea).

### Cloning of the pSpCas9 (BB)-2A-Puro plasmid

The DNA containing targeting sequence was inserted into the cloning site of the plasmid by using Bbs1 endonuclease. (Fig. S9). The cloned plasmid was transfected into MCF-7/ADR cells using Lipofectamine 3000.

### *In vitro* transcription

The sgRNA was synthesized by using HiScribe™ T7 High Yield RNA Synthesis Kit for 16 hr at 37 °C. The synthesized sgRNA was purified by isoamyl alcohol extraction.

### Expression and purification of Cas9

The pMJ806 plasmid was transformed into Rosetta™ 2 (DE3) Competent Cells. The transformed *E.coli* cells were cultured in 2X TY Medium. When optical density at 600 nm reached 0.5 ~ 0.6, IPTG (0.2 mM) was added to the culture, which was incubated further for 18 hr at 18 °C. Bacterial cells were lysed in lysis buffer (20 mM Tris-HCl, pH 7.9, 300 mM NaCl, 20 mM imidazole, 1XPIC, 200 μM PMSF, 10 μg/ml lysozyme). The lysate was sonicated on ice and ultra centrifuged at 13000 rpm for 1 hr at 4 °C. After that, the supernatant was bound to HisPur^TM^ Ni-NTA Resin by incubating for 1 hr at 4 °C. Resin was washed with washing buffer (20 mM Tris-HCl, pH 7.9, 300 mM NaCl, 20 mM Imidazole, and 2 mM PMSF). His-tagged MBP-Cas9 was eluted with elution buffer (20 mM Tris-HCl, pH7.9, 300 mM NaCl, 250 mM imidazole, and 2 mM PMSF). MBP fusion-tag was cleaved by TEV protease at 4 °C overnight. The solution containing Cas9 was concentrated by using Amicon Ultra-15 Centrifugal Filter Unit with Ultracel-100 membrane.

### *In vitro mdr1* cleavage by the Cas9-sgRNA complex

The *mdr1* sequence was obtained by PCR using the primers shown in Table S1 and genomic DNA of MCF-7/ADR cells as the template. The Cas9-sgRNA ribonucleoprotein complex was incubated with *mdr1* DNA (200 ng) for 2 hr at 37 °C. Then, the enzymatic reactions were examined on 2% agarose gel.

### Cell culture

MCF-7/ADR and MCF-7 cells were cultured in RPMI and DMEM including 10% FBS and antibiotics (1% penicillin and 1% streptomycin) at 37 °C with 5% CO_2_ in an incubator.

### Intracellular delivery of the Cas9-sgRNA complex

For delivery by using LF3K, LF3K (10 μL) was added to the mixture of Cas9 and sgRNA that was pre-incubated for 10 min at 37 °C^29^. For delivery by using PTD, Cas9 was conjugated with TAT peptide according to the previously reported protocol^22^, and mixed with sgRNA. All complex solutions were prepared by using 2:1 ratio of Cas9 and sgRNA. The prepared ribonucleoprotein complex was diluted in serum free RPMI (200 nM) and treated to the cells (1 × 10^5^) cultured on a 6-well plate for 4 hr. Then, the cells were washed with PBS, and fresh media was supplied. After cultivation for additional two days, the cells were harvested.

### T7E1 cleavage assay

Genomic DNA was extracted by using *MagListo*™ 5 M Genomic DNA Extraction Kit. The substrate sequence for T7E1 assays was amplified from the extracted DNA by using the primers (Table S1). The sequence (200 ng) in the reaction buffer (50 μL) supplied by the manufacturer was heated to 95 °C for 10 min. Then it was transferred to an 80 °C water bath and incubated there for 5 min, and then further cooled down to room temperature slowly for 45 min. To the solution was added T7E1 (20 μL), and the mixture was incubated for 15 min at 37 °C. The reactions were analyzed by gel electrophoresis using 1.2% agarose gel run in 0.5X TBE buffer at 70 V. The DNA bands on the gel were stained with SYBR Gold and visualized by INV-16 M Davinch *In vivo* Imaging System (Davinch K, Korea). Band intensity was analyzed by ImageJ.

### Western blot

Cells were harvested and lysed with RIPA lysis buffer. Proteins from the lysate (20 μg) was separated by gradient SDS-PAGE (10%), transferred to a positively charged membrane, and incubated with solutions containing anti-MDR1 (dilution factor 1:5000) and anti-β-actin (dilution factor 1:5000). The bands of MDR1 and β-actin were visualized by using SuperSignal™ West Pico Chemiluminescent and imaged by WSE-6100 LuminoGraph (ATTO, Japan).

### Cell viability assay

Cas9-sgRNA-treated cells were plated in 96-well (1 × 10^4^) and cultured in the medium containing 0 – 100 μM DOX at 37 °C for 24 hr in a 5% CO_2_ humidified incubator. The CCK-8 solution (10 μL) was added to each well, and the microwell plate was incubated at 37 °C for 4 hr in the incubator. The absorbance was then measured at 450 nm using Spectra MAX340 (Molecular devices, USA).

### Flow cytometry

Cas9-sgRNA-treated cells were plated in 12-well (4 × 10^4^) and cultured in the medium containing DOX (10 μM) at 37 °C for 6 hr in a 5% CO_2_ humidified incubator. Cells were pelleted and resuspended in an appropriate volume of DPBS and immediately subjected to a flow cytometry. The accumulation of DOX was quantified by Guava easyCyte™ Flow Cytometer (Merck Millipore, Germany).

### Fluorescence microscopy

Cas9-sgRNA-treated cells were plated in 6-well (1 × 10^5^) and cultured in the medium containing DOX (10 μM) at 37 °C for 6 hr in a 5% CO_2_ humidified incubator. The live cell imaging was performed by using a LSM 700 Axioobserver (Carl Zeiss, Germany).

## Additional Information

**How to cite this article**: Ha, J. S. *et al.* Overcoming doxorubicin resistance of cancer cells by Cas9-mediated gene disruption. *Sci. Rep.* **6**, 22847; doi: 10.1038/srep22847 (2016).

## Supplementary Material

Supplementary Information

## Figures and Tables

**Figure 1:**
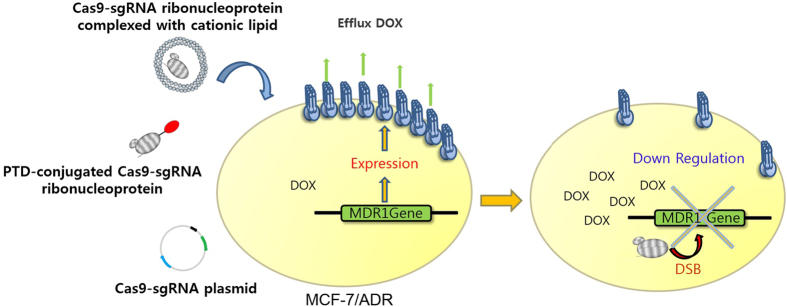
Schematic presentation of Cas9-sgRNA delivery system for down regulation of MDR1. Cas9-sgRNA system was introduced into the multidrug resistant cells (MCF-7/ADR) via transfection of the cells with the plasmid for expression of Cas9-sgRNA and intracellular delivery of the Cas9-sgRNA ribonucleoprotein complex using either a PTD peptide or Lipofectamine.
