# Supplementary material for: Overcoming doxorubicin resistance of cancer cells by Cas9-mediated gene disruption
Source: Sci Rep. 2016 Mar 10;6:22847. doi: 10.1038/srep22847 (PMC4785396; doi:10.1038/srep22847)
Supplement: Supplementary Information [file srep22847-s1.pdf]

Supplementary Information

Overcoming doxorubicin resistance of cancer cells by Cas9-mediated gene disruption

Jong Seong Ha[a,+], Juyoung Byun[b,+], and Dae-Ro Ahn[a,b,*]

a. The Center for Theragnosis, Biomedical Research Institute, Korea Institute of Science and Technology, Hwarangro 14-gil 5, Seongbuk-gu, Seoul 136-791, Republic of Korea

b. Department of Biological Chemistry, KIST School, University of Science and Technology (UST), Hwarangro 14-gil 5, Seongbuk-gu, Seoul 136-791, Republic of Korea

*Correspondence and requests for materials should be addressed to Dae-Ro Ahn (drahn@kist.re.kr)

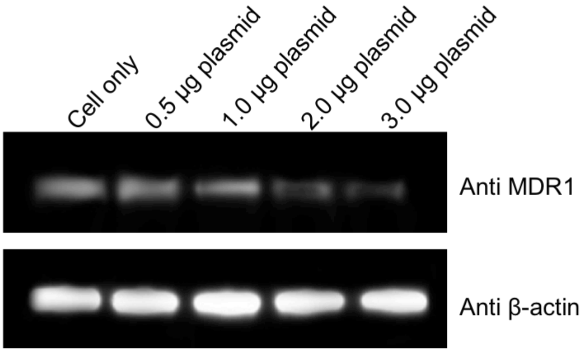

Figure S1. Western blot of MDR1 protein in MCF-7/ADR cells treated with the Cas9-sgRNA plasmid

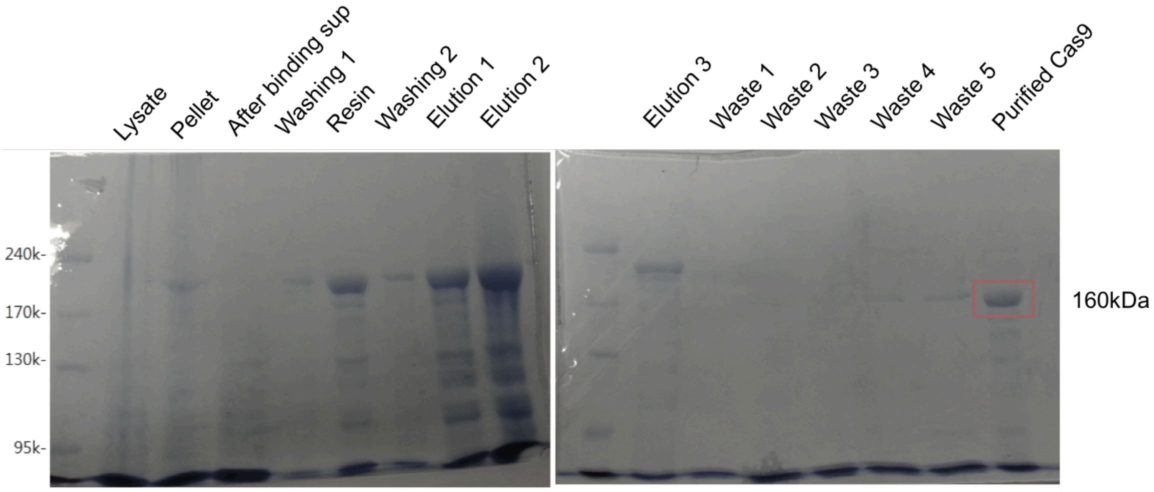

Figure S2. SDS-PAGE showing purification of recombinant Cas9

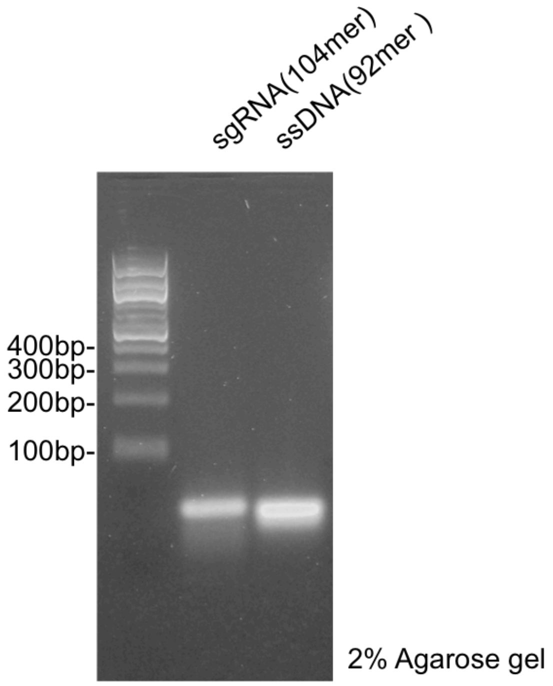

Figure S3. *In vitro* transcription of sgRNA targeting *mdr1*. Mobility of the single stranded RNA nucleotide was compared with that of a 92-mer single-stranded reference DNA as well as with that of double stranded DNA size markers.

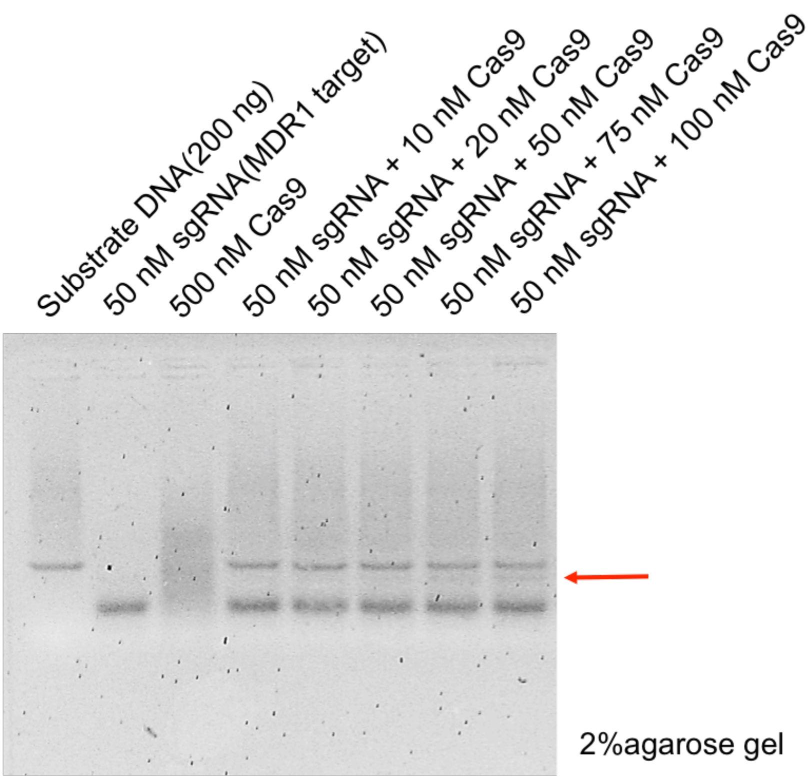

Figure S4. Screening optimal ratio between Cas9 and sgRNA to be used for preparation of the ribonucleoprotein complex.

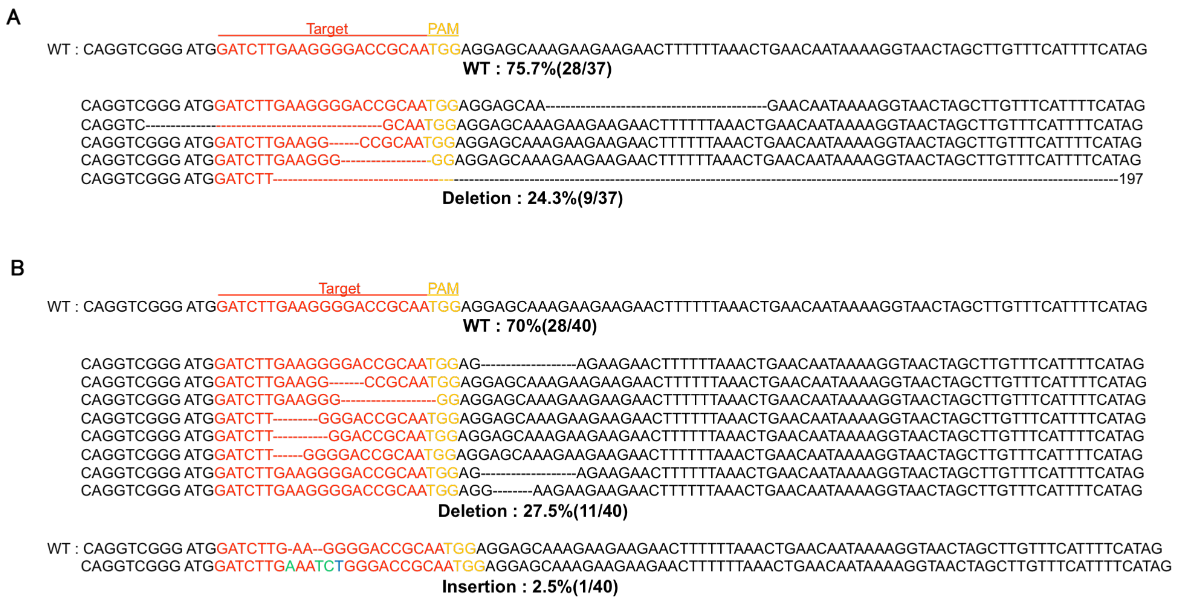

Figure S5. Indel patterns of the target region in *mdr1* resulting from treatment of (A) plasmid and (B) RNP complex using TAT. After the treatments, genomic DNA was extracted and subject to PCR to amplify the target region. The PCR product was inserted into TA cloning vector and transformed into DH5α bacterial cells. Plasmids from 50 colonies in each group were sequenced to reveal the indel patterns. 13 colonies from the plasmid-treated group and 10 colonies from the RNP complex-treated group contained the self-ligated TA cloning vector. Green letters indicate insertions. The blue letter indicates mutation. Red letters indicate target region. Orange letters indicate the PAM sequence.

Target PAM

Off-target 1 GGATGGCACTTGGCTCTTGAAGGGGACCCCACAGGCCTAGAAGGGGTCCCTACTTCCCTCCATTCCAGGCAGCAGATGTACTGG
GGGCGTGGGGACAGCAGAG

Target PAM

Off-target 2 TGAAAAGAATAAGATCTTGAAGGGCACAGGGAAGGGAGATCAGTGACAATTTAAGGGAGTACAGGGCCAGTAGATCATCTGATCA
AGACATAGAAAGTGTGTT

Figure S6. Off-target sites sharing sequences with the *mdr1* target were selected by using a on-line genome search tool (www.blueheronbio.com). Plasmids containing off-target regions prepared by using the same manner adopted for indel analysis in Figure S5 were sequenced to examine off-target activity of Cas9. Plasmids from 10 colonies in each group were sequenced. In the RNP complex-treated groups four colonies and one colony supposed to have the off-target 1 and the off-target 2 sequences, respectively, were found to have the self-ligated TA cloning vector. Except them, all plasmids contained the corresponding off-target inserts without any indel. Red letters indicate sequences shared with the *mdr1* target. Orange letters indicate the PAM sequence.

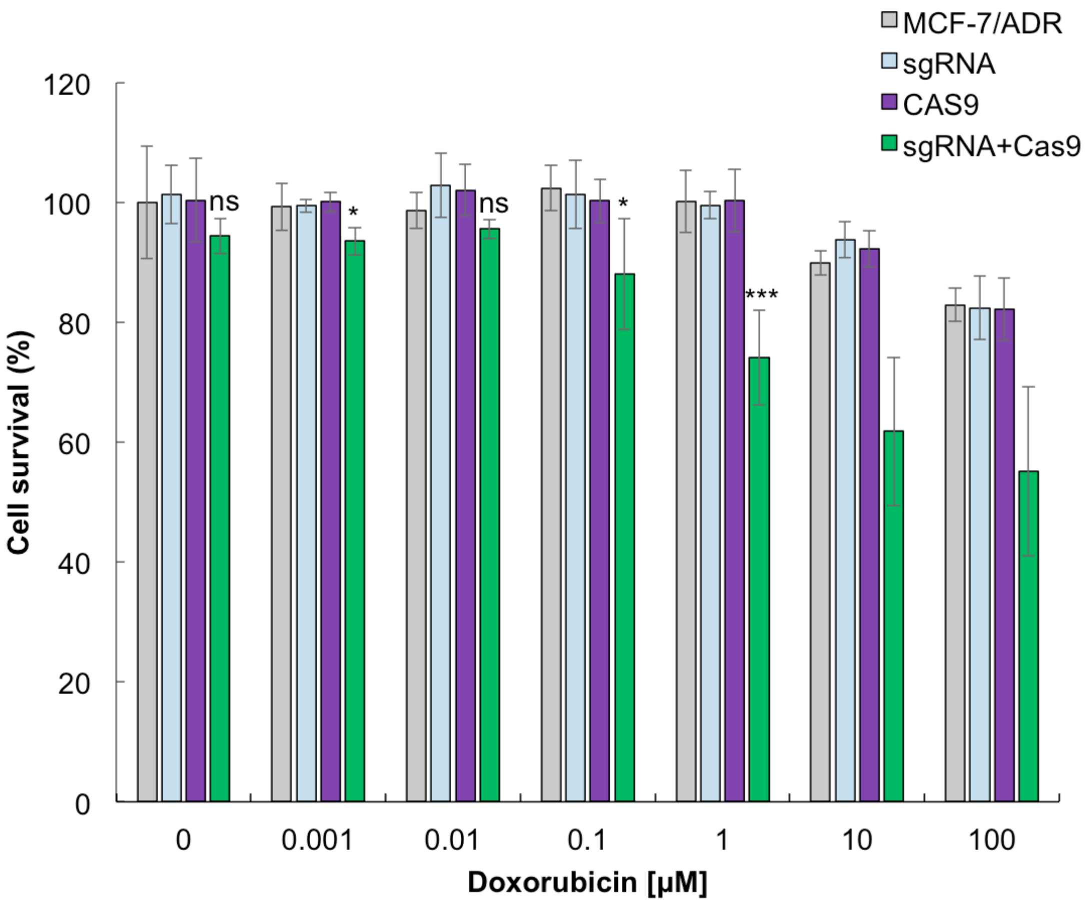

Figure S7. Cytotoxicity of doxorubicin in MCF-7/ADR cells treated with sgRNA or Cas9 alone. The data represent the mean ± s.d. (n=4) ns $P > 0.05$, *$P \leq 0.05$, **$P \leq 0.01$, ***$P \leq 0.001$ vs. untreated MCF-7/ADR cells

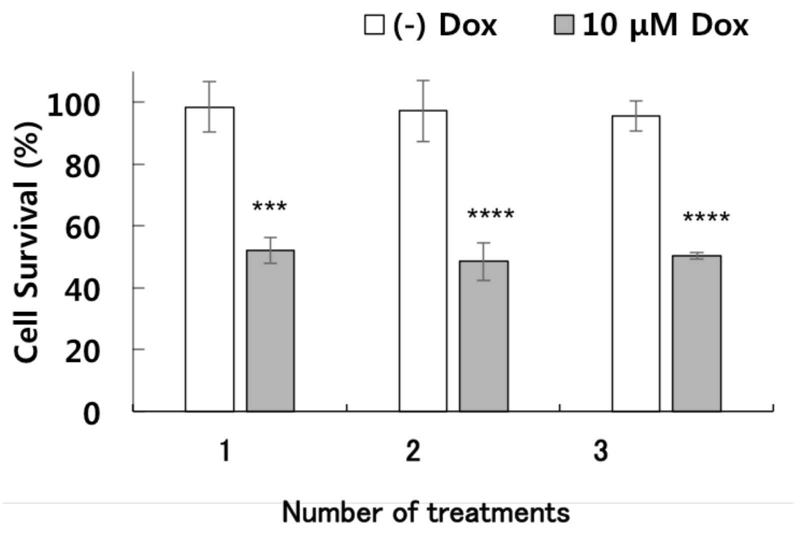

Figure S8. Cytotoxicity of doxorubicin in MCF-7/ADR cells repeatedly treated with the Cas9-sgRNA plasmid (1 to 3 times). The data represent the mean ± s.d. (n=4) ****P* ≤ 0.001 and *****P* ≤ 0.0001 vs. MCF-7/ADR cells in the absence of doxorubicin.

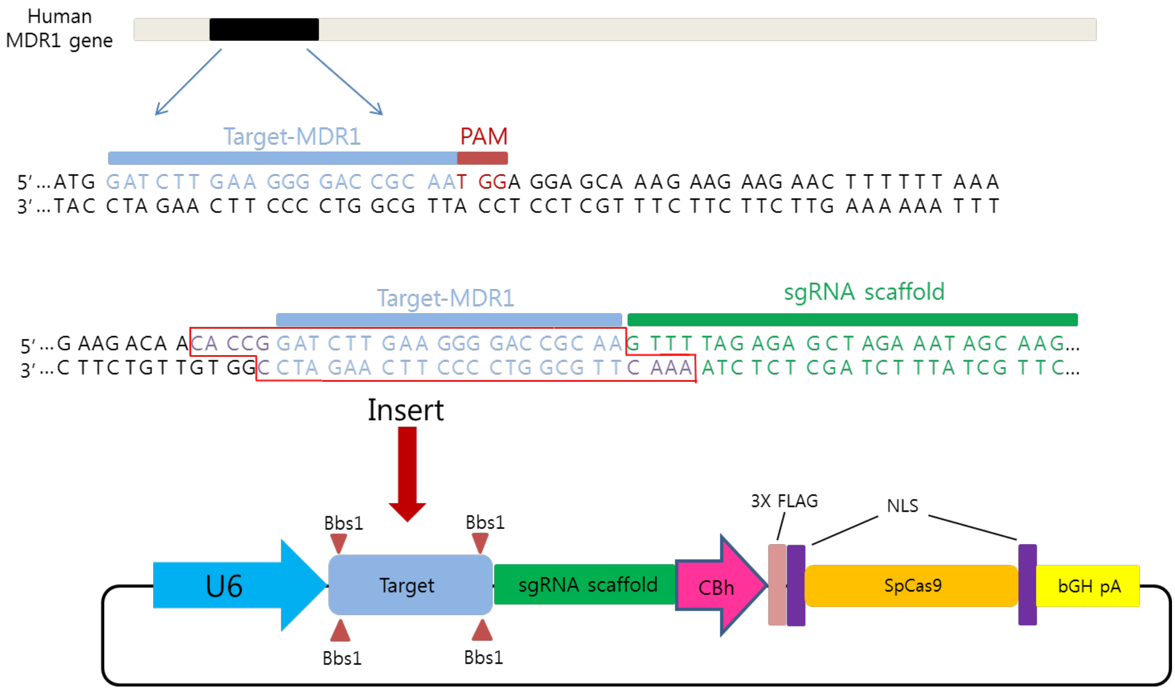

Figure S9. Design of *mdr1*-targeting sequence in sgRNA.

Table S1. The DNA sequences used in this study.

PCR primers for *In vitro* cleavage assays	Foward-5’GGTTAGTCTCACCTCCAG3’
	Reverse-5’AAAAAGTTCTTCTTCTTTGC3’
The *mdr1* target insert sequence	5'[Phos]CACCGGATCTTGAAGGGGACCGCAA3' (Sense)
	5'[Phos]AAACTTGCGGTCCCCTTCAAGATCC3' (Antisense)
DNA template for in vitro transcription of sgRNA targeting *mdr1*	5'GGATTCTAATACGACTCACTATAGGGATCTTGAAGGGGACCGCAA GTTTTAGAGCTAGAAATAGCAAGTTAAAATAAGGCTAGTCCGTTATCA ACTTGAAAAAGTGGCACCGAGTCGGTGCTTTTTTT3' (Sense)
	5'AAAAAAAGCACCGACTCGGTGCCACTTTTTCAAGTTGATAACGGA CTAGCCTTATTTTAACTTGCTATTTCTAGCTCTAAAACTTGCGGTCCC CTTCAAGATCCCTATAGTGAGTCGTATTAGAATCC3' (Antisense)
PCR primers for T7E1 cleavage assays	Foward-5’TCTTACTGCTCTCTGGCTTCGA3’
	Reverse-5’CTTGATCATTACACATTCCGGGCATGT3’
